# iNOS as a Driver of Inflammation and Apoptosis in Mouse Skeletal Muscle after Burn Injury: Possible Involvement of Sirt1 S-Nitrosylation-Mediated Acetylation of p65 NF-κB and p53

**DOI:** 10.1371/journal.pone.0170391

**Published:** 2017-01-18

**Authors:** Harumasa Nakazawa, Kyungho Chang, Shohei Shinozaki, Takashi Yasukawa, Kazuhiro Ishimaru, Shingo Yasuhara, Yong-Ming Yu, J. A. Jeevendra Martyn, Ronald. G. Tompkins, Kentaro Shimokado, Masao Kaneki

**Affiliations:** 1 Department of Anesthesia, Critical Care and Pain Medicine, Massachusetts General Hospital, Harvard Medical School, Charlestown, Massachusetts, United States of America; 2 Shriners Hospitals for Children, Boston, Massachusetts, United States of America; 3 Department of Geriatrics and Vascular Medicine, Tokyo Medical and Dental University Graduate School, Tokyo, Japan; 4 Department of Surgery, Massachusetts General Hospital, Harvard Medical School, Boston, Massachusetts, United States of America; National Institutes of Health, UNITED STATES

## Abstract

Inflammation and apoptosis develop in skeletal muscle after major trauma, including burn injury, and play a pivotal role in insulin resistance and muscle wasting. We and others have shown that inducible nitric oxide synthase (iNOS), a major mediator of inflammation, plays an important role in stress (e.g., burn)-induced insulin resistance. However, it remains to be determined how iNOS induces insulin resistance. Moreover, the interrelation between inflammatory response and apoptosis is poorly understood, although they often develop simultaneously. Nuclear factor (NF)-κB and p53 are key regulators of inflammation and apoptosis, respectively. Sirt1 inhibits p65 NF-κB and p53 by deacetylating these transcription factors. Recently, we have shown that iNOS induces S-nitrosylation of Sirt1, which inactivates Sirt1 and thereby increases acetylation and activity of p65 NF-κB and p53 in various cell types, including skeletal muscle cells. Here, we show that iNOS enhances burn-induced inflammatory response and apoptotic change in mouse skeletal muscle along with S-nitrosylation of Sirt1. Burn injury induced robust expression of iNOS in skeletal muscle and gene disruption of iNOS significantly inhibited burn-induced increases in inflammatory gene expression and apoptotic change. In parallel, burn increased Sirt1 S-nitrosylation and acetylation and DNA-binding capacity of p65 NF-κB and p53, all of which were reversed or ameliorated by iNOS deficiency. These results indicate that iNOS functions not only as a downstream effector but also as an upstream enhancer of burn-induced inflammatory response, at least in part, by Sirt1 S-nitrosylation-dependent activation (acetylation) of p65 NF-κB. Our data suggest that Sirt1 S-nitrosylation may play a role in iNOS-mediated enhanced inflammatory response and apoptotic change, which, in turn, contribute to muscle wasting and supposedly to insulin resistance after burn injury.

## Introduction

Metabolic dysfunction in skeletal muscle is a major complication after major trauma, including burn injury, and negatively affects the clinical outcome of patients with major trauma [1, 2]. These metabolic alterations include insulin resistance, hyperglycemia, hypermetabolism, increased lactate production, catabolism and muscle wasting. Inflammatory response plays a critical role in obesity- and stress (e.g., burn)-induced insulin resistance [3–5]. Insulin resistance and apoptotic change contribute to muscle wasting [6–9]. Despite intensive investigation for many years, it is not fully understood how inflammation is initiated and sustained after major trauma and how inflammation causes insulin resistance.

Inducible nitric oxide synthase (iNOS) is a major downstream mediator of inflammation in various cell types including skeletal muscle cells. We and others have previously shown that inhibition of iNOS by gene disruption or pharmacological inhibitors ameliorates obesity- and stress (i.e., burn, lipopolysaccharide [LPS])-induced insulin resistance in rodents [10–14]. Burn injury suppresses insulin-stimulated glucose uptake by skeletal muscle compared with sham-burn in wild-type mice at 3 days after burn, which parallels impaired insulin-stimulated phosphorylation of insulin signaling molecules, insulin receptor, insulin receptor substrate-1, Akt and glycogen synthase kinase-3β [13]. iNOS deficiency almost completely prevents or significantly ameliorates burn-induced inhibition of response to insulin in term of glucose uptake and phosphorylation of the insulin signaling molecules in mouse muscle [13]. Of note, the induction of iNOS expression and the development of insulin resistance are time-dependent and parallel with each other after burn injury [13]. The maximum effects of burn injury on both iNOS expression and insulin resistance were observed at 3 days after burn injury in mice [13]. These data clearly indicate the close link between the iNOS induction and the development of insulin resistance after burn. Nonetheless, the precise mechanisms by which iNOS induces and/or exacerbates stress (e.g., burn)- and obesity-induced insulin resistance are not well understood.

Protein S-nitrosylation is the covalent attachment of nitric oxide (NO) moiety to reactive cysteine thiols and a major mediator of physiological and pathophysiological actions of NO [15]. Our recent study has demonstrated that iNOS-mediated S-nitrosylation of Sirt1, a NAD^+^-dependent deacetylase, inactivates Sirt1 and thereby increases acetylation and activity of p65 (aka RelA) subunit of nuclear factor (NF)-κB and p53 [16]. The prototypic form of NF-κB is a heterodimer consisting p65 and p50 or p52 subunits. p65 NF-κB and p53 are key transcription factors in the regulation of inflammatory response and apoptosis, respectively. NF-κB upregulates the transcription of iNOS and pro-inflammatory molecules, including interleukin (IL)-1β, tumor necrosis factor (TNF)-α and toll-like receptor (TLR)-4. In addition, NF-κB activation induces muscle wasting by increasing the transcription of muscle-specific ubiquitin ligases (aka atrogenes), muscle ring-finger 1 (Murf1) and atrogin-1 (aka muscle atrophy F-box [MAFbx]), in skeletal muscle [17, 18]. p53 activation causes apoptosis by upregulating expression of the pro-apoptotic genes, including Bax, Fas and Fas ligand (FasL).

Sirt1 inhibits p65 NF-κB and p53 by deacetylation, reducing inflammation and apoptosis [19–22]. Conversely, Sirt1 inactivation results in activation of p65 NF-κB and p53 by increasing acetylation of the transcription factors [19–22]. We have shown that in rodent models of LPS-induced systemic inflammation and age-related muscle wasting, increased S-nitrosylation of Sirt1 and the associated activation of p65 NF-κB and p53 require the activity of iNOS. In cultured COS-7 cells, Sirt1 is required for NO donor to increase acetylation of p65 NF-κB and p53, indicating that Sirt1 inactivation plays an important role in NO-induced acetylation of these transcription factors [16]. Collectively, these data indicate that iNOS can function as an upstream enhancer of inflammatory response as well as a downstream target gene of NF-κB.

Host response to major trauma includes inflammation, apoptosis and metabolic alterations. In fact, burn injury causes inflammatory response and apoptotic change as well as insulin resistance and muscle wasting in burn patients and mice [23–27]. Little is known, however, about the interrelation between these pathophysiological processes that take place simultaneously. Although p65 NF-κB and p53 are involved in these pathophysiological processes, acetylation or activity of p65 NF-κB and p53 has not been well studied in major trauma including burn injury. The effect of burn injury on Sirt1 is not known. Moreover, the role of iNOS in inflammation and apoptosis after major trauma, including burn injury, has not been investigated, whereas iNOS plays an important role in burn-induced insulin resistance [13]. To investigate the relation between inflammation, apoptosis and metabolic alteration, we studied the role of iNOS in burn-induced inflammatory response and apoptotic change in mouse skeletal muscle and the effects of burn on acetylation and activation of p65 NF-κB and p53, Sirt1 S-nitrosylation.

## Materials and Methods

### Ethics statement

All experiments were carried out in accordance with the institutional guidelines and the study protocol was approved by the Institutional Animal Care and Use Committee (IACUC) at the Massachusetts General Hospital (the protocol #: 2007N000020). The animal care facility is accredited by the Association for Assessment and Accreditation of Laboratory Animal Care.

### Animals

Male C57BL/6 and iNOS knockout mice on C57BL/6 background (Jackson Laboratory, Bar Harbor, ME) were used at 8 weeks of age in this study. All the mice were housed in a controlled environment (20–22°C; 12 hours of light/dark) from seven days prior to the burn injury or sham-burn injury procedure through the end of the study. The mice were provided with standard rodent chow and water ad libitum. The pair-feeding was performed as described previously [13]. A full-thickness burn injury comprising 30% of total body surface area was produced under anesthesia with pentobarbital sodium (50 mg/kg BW, IP) by immersing the abdomen for 6 sec and both sides of the flank for 4 sec in 80°C water as previously described [13, 25]. Sham-burned mice were immersed in lukewarm water. To minimize animal suffering and distress after burn injury, buprenorphine (0.1 mg/kg BW, SC) was administered 30 min prior to burn and sham-burn and every 8 h for 72 h after burn or sham-burn. For resuscitation, prewarmed normal saline (0.04 ml/g BW, IP) was injected just after burn or sham-burn. Postoperatively, the mice were kept warm with heating lamps and monitored continuously until recovery of consciousness and mobility. Signs of pain and/or distress (as indicated by reduced activity, change in temperament, decreased food intake, abnormal vocalization, abnormal posture, self-mutilation of wound) and mobility of the mice were monitored every 8 h for 72 h after burn or sham-burn and once daily thereafter until the end of the study. The body weight and food intake were measured every day. None of the mice died or showed any of the signs of pain or distress, 15% or greater body weight loss compared with the initial body weight just after burn or sham-burn, or skin wound infection in this study. 3 or 7 days after burn or sham-burn, rectus abdonimis and plasma were collected for biochemical analyses. At the end of study, the mice were euthanized by carbon dioxide asphyxiation.

### Immunoblot analysis

Immunoblotting was performed as previously described [28]. Briefly, muscle tissues were pulverized under liquid nitrogen and homogenized in homogenization buffer A (50 mM HEPES pH 8.0, 150 mM NaCl, 2 mM EDTA, 7.5% SDS, 2% CHAPS, 10% glycerol, 10 mM sodium fluoride, 2 mM sodium vanadate, 1 mM PMSF, 10 mM sodium pyrophosphate, protease inhibitor cocktail, 5 μM trichostatin A, and 10 mM nicotinamide). Trichostatin A and nicotinamide were added to prevent deacetylation during the experimental procedure. For the detection of acetylated and total NF-κB p65 and p53 from muscle tissues, to completely solubilize proteins including those in the nuclear and chromatin fractions, the pulverized samples were homogenized in ice-cold hypotonic buffer (20 mM HEPES pH 7.5, 10 mM MgCl_2_, 10 mM KCl, 10 mM sodium fluoride, 1 mM PMSF, 2 mM EDTA, 1 mM DTT, 20% glycerol, protease inhibitor cocktail, 5 μM trichostatin A, and 10 mM nicotinamide) for 15 min on ice, and the samples were centrifuged at 13,000g for 10 min at 4°C. Then, the precipitates were homogenized in homogenization buffer A. Equal amounts of protein, determined by detergent-compatible protein assay kit (Bio-Rad, Herculues, CA), were boiled for 5 min in Laemmli sample buffer, separated by SDS-PAGE, and transferred onto nitrocellulose membranes (Bio-Rad). The membranes were soaked in blocking buffer (GE Healthcare, Pittsburgh, PA) for 1 h and then incubated overnight at 4°C with anti-glyceraldehyde 3-phosphate dehydrogenase (GAPDH) (Trevigen, Gaithersburg, MD, #2275-PC-1) (dilution 1:25,000), anti-p65 NF-κB (#4747) (1:2,000), anti-acetylated p53 (Lys379; #2570) (1:2,000), anti-histone H3 (#9715) (1:10,000), anti-cleaved caspase-3 (#9664) (1:1,000), anti-NOS (pan) (#2977) (1:1,000) (Cell Signaling Technology, Danvers, MA), anti-acetylated p65 NF-κB (Lys310) (ab19870) (1:2,000), anti-phospho-p53(S15) (ab1431) (1:5,000), anti-phospho-p65 (S276) (ab106129) (1:5,000) (Abcam, Cambridge, MA), anti-p53 (sc-6243) (1: 2,000), anti-nNOS (sc-648) (dilution 1:5,000), anti-phospho-p65 (S311) (sc-33039) (1:5,000) (Santa Cruz Biotechnology, Santa Cruz, CA), anti-iNOS (#06–573) (1: 5,000) or anti-Sirt1 (#07–131) antibody (Millipore, Billerica, MA) (1:5,000), followed by incubation with secondary antibody (antibody recognizing rabbit or mouse IgG) conjugated to horse radish peroxidase for 1 h at room temperature (1:25,000 or 1:50,000). Antigen-antibody complexes were detected by enhanced chemiluminescence reagent (Lumigen, Southfield, MI). The immunoblots were scanned using the HP Scanjet 4850 (Hewlett-Packard, Palo Alto, CA). Densitometric analysis of the results was carried out using NIH Image software (ver. 1.62).

### Measurement of DNA-binding of p65 NF-κB and p53

DNA-binding capacity of p65 NF-κB and p53 were measured by TransAM p65 NF-κB and p53 Transcription Factor Assay kits (Active Motif, Carlsbad, CA), respectively.

### Detection of S-nitrosylated Sirt1

To detect S-nitrosylation of Sirt1, the biotin-switch assay was performed as previously described [16, 29]. Briefly, muscle tissues were homogenized in homogenization buffer B (PBS-HCl pH 3.5, 150 mM NaCl, 1 mM EDTA, 1 mM diethylenetriamine pentaacetic acid [DTPA], 7.5% SDS, 2% CHAPS, 0.1 mM neocuproine, 80 μM carmustine, 1 mM PMSF, protease inhibitor cocktail). After centrifugation, supernatants were mixed with 2 volumes of blocking buffer (PBS-HCl pH 3.5, 150 mM NaCl, 1 mM EDTA, 1 mM DTPA, 0.1 mM neocuproine, 30 mM methyl methanethiosulfonate [MMTS]) and incubated at 50°C for 20 min with vortex every 2 min. After removing excess MMTS, S-nitrosylated thiols were biotinylated with N-(6-[biotinamido]hexyl)-3’-(2’-pyridyldithio)-propionamide (HPDP-biotin) (0.2 mM) (Pierce, Waltham, MA) in the presence of sodium ascorbate (4 mM). After precipitation with −20°C acetone, streptavidin-agarose beads (Sigma) were added, and the samples were incubated for 1 h at room temperature. The beads were washed with HEN buffer (25 mM HEPES PH 7.5, 1 mM EDTA, 0.5 M NaCl) containing 0.5% NP-40, and proteins were eluted by incubation with elution buffer (20 mM HEPES pH 7.7, 1 mM EDTA, 100 mM NaCl, 200 mM DTT) for 30 min and separated by SDS-PAGE followed by immunoblotting with anti-SIRT1 antibody.

### Isolation of total RNA and quantitative reverse transcription polymerase chain reaction (RT-PCR)

Total RNA was isolated with TRIzol reagent (Life Technologies, Grand Island, NY) from skeletal muscle at 3 days after burn or sham-burn injury. The first-strand cDNA was synthesized from 1 μg of total RNA using the High Capacity cDNA Reverse Transcription Kit (Applied Biosystems, Waltham, MA). RT-PCR was performed using 10 ng of cDNA and TaqMan probes (Applied Biosystems) for IFN-γ (Mm01168134_m1), IL-1β (Mm99999061_mH), TNF-α (Mm00443258_m1), Bax (Mm00432051_m1), Fas (Mm01204974_m1), FasL (Mm00438864_m1), TLR-4 (Mm00445273_m1), atrogin-1 (Mm00499523_m1), Murf-1 (Mm01185221_m1), and 18S ribosomal RNA (Hs99999901_s1), conducted with Mastercycler ep realplex (Eppendorf, Hamburg, Germany). mRNA levels were normalized to that of 18S ribosomal RNA.

### Measurement of plasma HMGB1 concentration

Concentrations of HMGB1 in heparinized plasma were measured at 3 days after burn or sham-burn using a commercial kit for HMGB1 (Shino-Test Corporation, Tokyo, Japan).

### Cell death detection ELISA

To quantify an apoptotic change, cytoplasmic DNA fragments were evaluated as previously described [24]. Muscle tissues were homogenized in TEET buffer (5 mM Tris-HCl pH 8.0, 20 mM EDTA, 0.1% Triton X-100). After adjustment of protein concentrations, PEG8000 and NaCl were added to a final concentration of 2.5% and 1 M, respectively. The mixture was incubated for 10 min on ice and then centrifuged at 16,000 g for 10 min at 4°C. Cytosolic mono- and oligonucleosomes in the supernatants were quantified using Cell Death Detection ELISA Assay Plus (Roche, Basel, Switzerland).

### Morphometric analysis

To evaluate the effects of iNOS deficiency on burn-induced muscle wasting, which results from cumulative daily muscle mass loss, we studied the cross-sectional area of skeletal muscle at 7 days after burn or sham-burn, at the time point when burn induced a significant muscle wasting compared with sham-burn in our pilot study. At 7 days after burn or sham-burn, muscle samples were covered in OCT compound, frozen in liquid nitrogen, and cross-sectioned (4-μm thick). Mid-area slices were stained with hematoxylin and eosin (HE) staining to evaluate the myofiber cross-sectional area as previously described [6, 30].

### Statistical analysis

The data were compared with one-way ANOVA followed by Newman Keuls multiple comparison test. A value of p<0.05 was considered statistically significant. All values are expressed as means ± SEM.

## Results

### Time-dependent induction of iNOS paralelled acetylation of p65 NF-κB and p53 after burn injury

**Fig 1:**
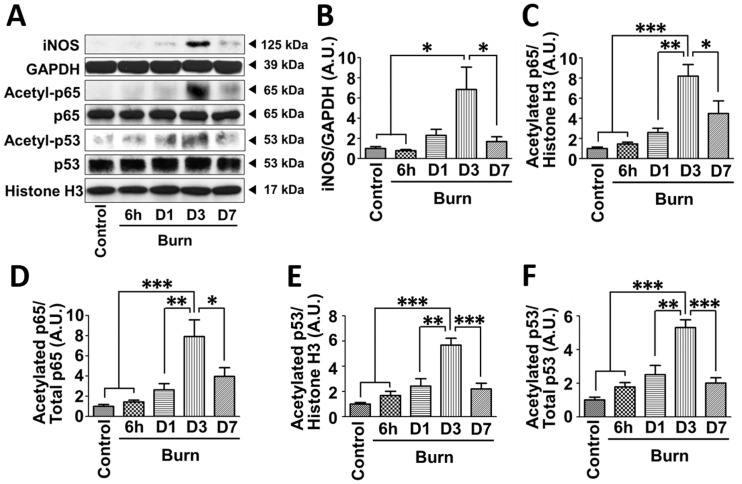
iNOS induction paralleled acetylation of p65 NF-κB and p53 in skeletal muscle of burned mice. iNOS protein expression and acetylation of p65 NF-κB and p53 were examined in skeletal muscle of naïve (Control) mice and at 6 h, 1, 3 and 7 days after burn. iNOS protein expression and acetylation of p65 NF-κB and p53 were significantly increased at 3 days after burn. *P<0.005, **P<0.01, ***P<0.001. n = 4 mice per group.

Consistent with our previous studies [13, 25], burn injury increased iNOS expression in a time-dependent manner in mouse muscle and the maximum level of iNOS expression was observed at 3 days after burn (Fig 1A and 1B). In parallel with iNOS induction, acetylation (activation) of p65 NF-κB and p53 was significantly increased at 3 days after burn injury compared with naïve control mice (Fig 1A, 1C–1F). At 1 and 7 days after burn, iNOS expression and acetylation of p65 NF-κB and p53 appeared to be increased relative to naïve control mice, but there were no statistically significant differences. Protein expression of p65 NF-κB, p53, GAPDH and histone H3 was not significantly altered by burn injury (S1 Fig).

### iNOS deficiency reversed burn-induced acetylation and DNA-binding of p65 NF-κB and p53 in burned mice

**Fig 2:**
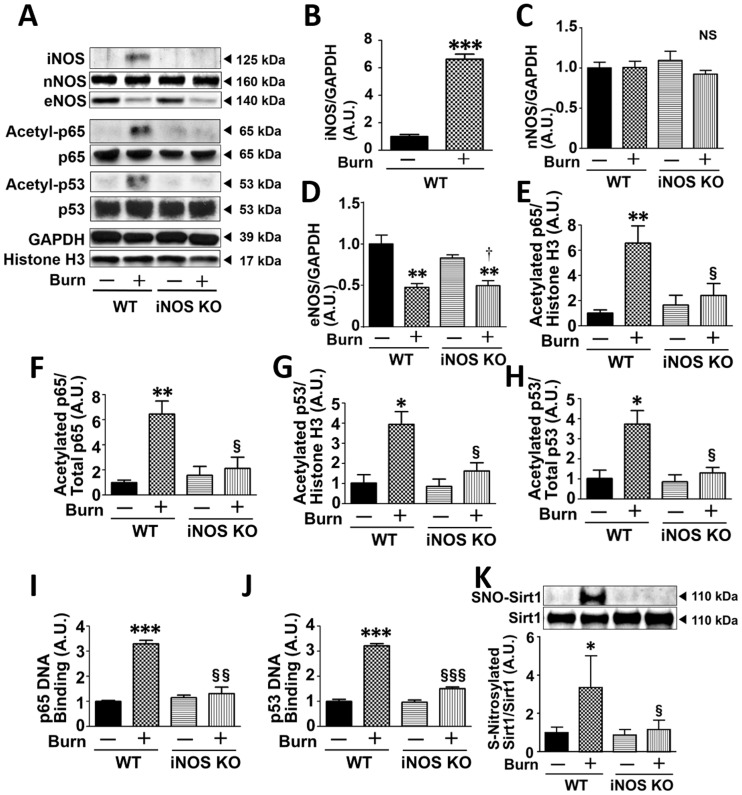
iNOS deficiency inhibited burn-induced increased acetylation and DNA-binding capacity of p65 NF-κB and p53, and S-nitrosylation of Sirt1 in mouse skeletal muscle. Burn injury significantly increased iNOS expression in wild-type mice (WT), but not iNOS knockout mice (iNOS KO), at 3 days post-burn. Neither burn injury nor iNOS deficiency altered nNOS expression. eNOS expression was significantly decreased at 3 days post-burn both in WT and iNOS KO mice to a similar extent. There was no significant difference in eNOS expression between WT and iNOS KO mice. Acetylation and DNA-binding capacity of p65 NF-κB and p53 and Sirt1 S-nitrosylation (SNO-Sirt1) were significantly increased in WT at 3 days post-burn, but not in iNOS KO. *P<0.05, **P<0.01, ***P<0.001 vs. WT-Sham and iNOS KO-Sham, §P<0.05, §§P<0.01, §§§P<0.001 vs. WT-Burn. n = 3 mice per group for Sham; n = 5 mice per group for Burn.

We have previously shown that iNOS deficiency inhibits burn-induced insulin resistance [13], but the role of iNOS in inflammation and apoptosis has not been studied in burn or major trauma. To investigate the role of iNOS in acetylation (activation) of p65 NF-κB and p53, we studied the effects of iNOS deficiency at 3 days post-burn injury. iNOS expression was increased in wild-type mice, but not iNOS KO mice, at 3 days after burn injury (Fig 2A and 2B). On the other hand, neither burn injury nor iNOS deficiency altered protein expression of neuronal NO synthase (nNOS) in mouse skeletal muscle (Fig 2A and 2C). Burn injury decreased endothelial NO synthase (eNOS) expression both in wild-type and iNOS KO mice to a similar extent (Fig 2A and 2D). After sham-burn injury, there was no difference in eNOS expression between wild-type and iNOS KO mice.

In wild-type mice, burn injury increased acetylation of p65 NF-κB and p53 in skeletal muscle compared with sham-burned mice. In contrast, iNOS deficiency prevented burn-induced acetylation of p65 NF-κB (Fig 2A, 2E and 2F) and p53 (Fig 2A, 2G and 2H). Protein expression of GAPDH, p65 NF-κB, p53 and histone H3 was not significantly altered by burn or iNOS deficiency (S2 Fig).

Consistent with increased acetylation of p65 NF-κB and p53 by burn and its reversal by iNOS deficiency, burn injury increased DNA-binding capacity of p65 NF-κB and p53, which was almost completely reversed by iNOS deficiency (Fig 2I and 2J). iNOS deficiency did not alter acetylation or DNA-binding capacity of p65 NF-κB and p53 in sham-burned mice.

### Burn-induced acetylation of p65 NF-κB and p53 was associated with iNOS-dependent S-nitrosylation of Sirt1

Sirt1 deacetylates p65 NF-κB and p53 and thereby inhibits the activity of these transcription factors [19, 21, 22]. Our previous study has shown that iNOS-mediated S-nitrosylation of Sirt1 inactivates the deacetylase, leading to increased acetylation and activation of p65 NF-κB and p53 [16]. Burn injury increased S-nitrosylation of Sirt1 compared with sham-burn, which was reversed by iNOS deficiency (Fig 2K). Protein expression of Sirt1 was not altered by burn or iNOS deficiency (Fig 2K and S2 Fig).

### iNOS deficiency did not alter burn-induced phosphorylation of p65 NF-κB and p53

**Fig 3:**
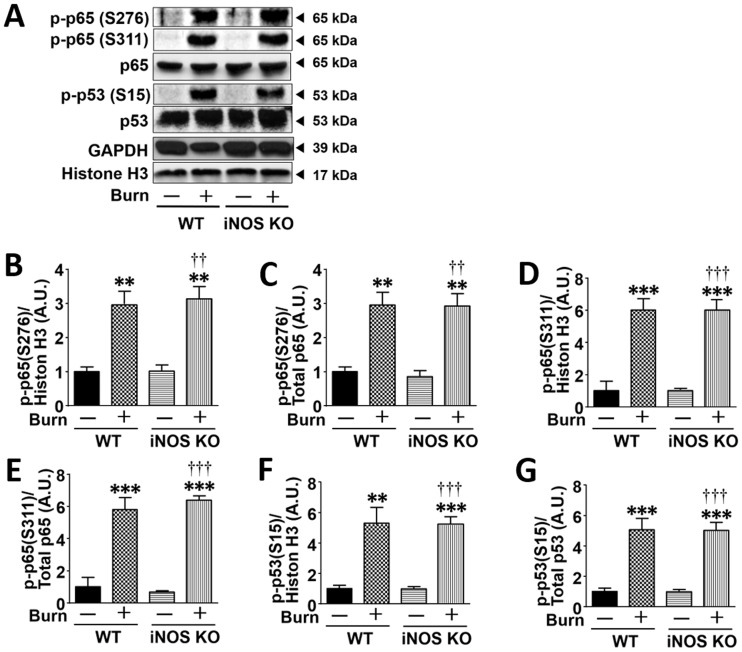
iNOS deficiency did not alter burn-induced phosphorylation of p65 NF-κB and p53 in mouse skeletal muscle. Phosphorylation of p65 NF-κB and p53 were significantly increased in mouse skeletal muscle at 3 days after burn injury compared with sham-burn. However, iNOS deficiency did not alter phosphorylation of p65 NF-κB and p53 in sham-burned and burned mice. *P<0.05, **P<0.01, ***P<0.001 vs. WT-Sham, †P<0.05, ††P<0.01, †††P<0.001 vs. iNOS KO-Sham. n = 3 mice per group for Sham; n = 5 mice per group for Burn.

In addition to acetylation, the transcriptional activities of p65 NF-κB and p53 are also regulated by phosphorylation [31–33]. Burn injury increased phosphorylation of p65 NF-κB and p53 in mouse skeletal muscle. Unlike acetylation, iNOS deficiency did not alter burn-induced phosphorylation of p65 NF-κB and p53 (Fig 3A–3G).

### iNOS deficiency inhibited expression of the downstream target genes of p65 NF-κB and p53

**Fig 4:**
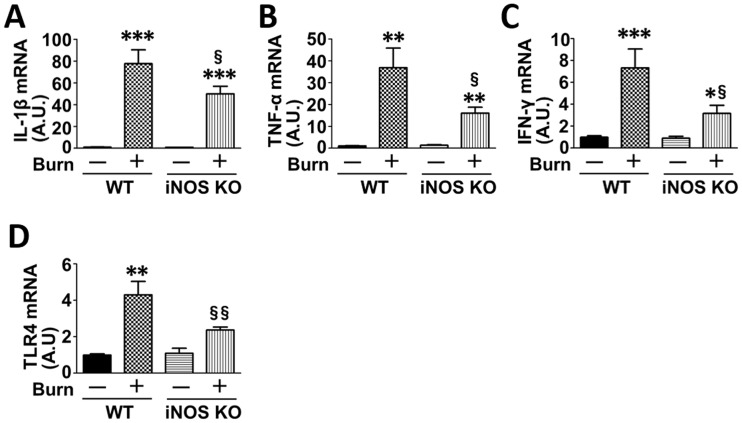
Effects of burn and iNOS deficiency on mRNA levels of inflammatory genes in mouse muscle. At 3 days after burn or sham-burn, mRNA levels of IL-1β, TNF-α, IFN-γ and TLR-4 were increased by burn in wild-type (WT) mice. Burn-induced increase in mRNA levels of these genes was attenuated in iNOS knockout (iNOS KO) mice. **P<0.01, ***P<0.001 vs. WT-Sham and iNOS KO-Sham, §P<0.05, §§P<0.01 vs. WT-Burn. n = 3 mice per group for Sham; n = 5 mice per group for Burn.

**Fig 5:**
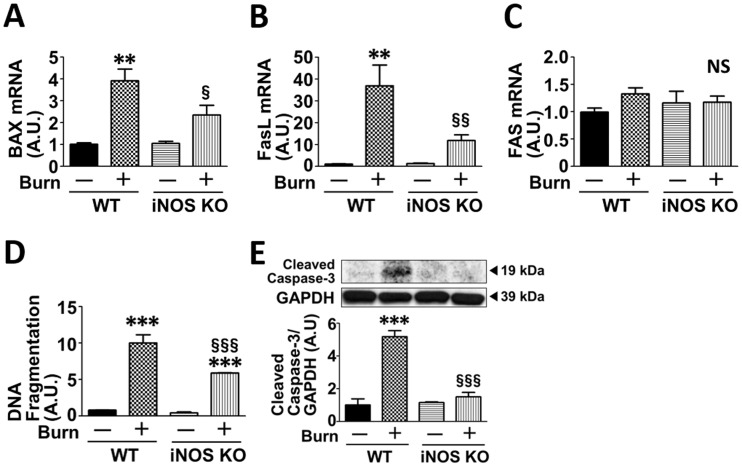
Burn-induced apoptotic change was mitigated by iNOS deficiency. At 3 days after burn or sham-burn, mRNA levels of Bax and FasL, DNA fragmentation and cleaved caspase-3 were increased by burn injury in wild-type (WT) mice, all of which were mitigated in iNOS knockout (iNOS KO) mice. mRNA levels of Fas was not significantly altered by burn or iNOS deficiency. **P<0.01, ***P<0.001 vs. WT-Sham and iNOS KO-Sham, §P<0.05, §§P<0.01, §§§P<0.001 vs. WT-Burn, NS: not significant. n = 3 mice per group for Sham; n = 5 mice per group for Burn.

Next, we examined the effects of burn and iNOS deficiency on mRNA levels of inflammatory (IL-1β, TNF-α, IFN-γ and TLR-4) and pro-apoptotic molecules (Bax, Fas and FasL) that are transcriptionally upregulated by p65 NF-κB and p53, respectively. mRNA levels of IL-1β, TNF-α, IFN-γ, TLR-4, Bax and FasL were significantly increased at 3 days after burn injury compared with sham-burn. Burn-induced increase in the expression of these genes was significantly attenuated in iNOS knockout mice compared with burned wild-type mice (Figs 4A–4D, 5A and 5B). Although there appears to be a trend toward increased Fas mRNA level in burned wild-type mice, mRNA expression of Fas was not significantly altered by burn or iNOS deficiency (Fig 5C). iNOS deficiency did not alter mRNA expression of none of the genes tested in sham-burned mice.

### iNOS knockout mice were resistant to burn-induced apoptotic and atrophic changes in mouse skeletal muscle

To further investigate the impact of iNOS-dependent activation of p53 and p65 NF-κB, we examined the effects of burn on apoptotic change and muscle fiber size in wild-type and iNOS knockout mice. Consistent with previous studies [24, 34], burn increased apoptotic change in skeletal muscle, as judged by apoptotic DNA fragmentation and cleavage (activation) of caspase-3, at 3 days after post-burn, as compared with sham-burn. Burn-induced apoptotic changes were ameliorated by iNOS deficiency (Fig 5D and 5E).

**Fig 6:**
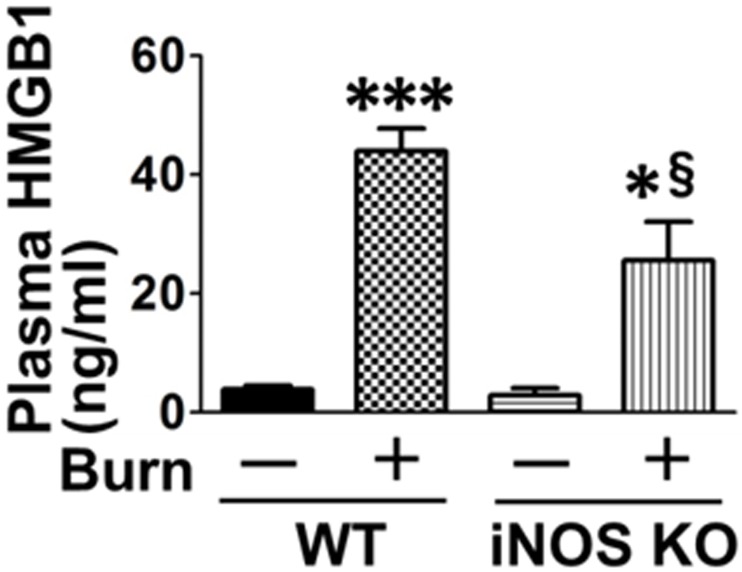
iNOS deficiency ameliorated burn-induced increase in circulating HMGB1. At 3 days after burn or sham-burn, plasma HMGB1 concentrations were significantly increased by burn injury in wild-type (WT) mice, which was mitigated in iNOS knockout (iNOS KO) mice. *P<0.05, ***P<0.001 vs. WT-Sham and iNOS KO-Sham, §P<0.05 vs. WT-Burn. n = 3 mice per group for Sham; n = 5 mice per group for Burn.

Damage-associated molecular patterns (DAMPs), such as high-mobility group box 1 (HMGB1), have been identified as a major contributor to sterile inflammation after major trauma [35]. Increased extracellular DAMPs including HMGB1 is linked to inflammation and apoptotic cell death [36], and NF-κB and p53 are involved in release of HMGB1 to the extracellular space [37–39]. We, therefore, measured circulating HMGB1 levels. Burn increased plasma HMGB1 concentration at 3 days post-burn compared with sham-burn. Burn-induced increased plasma HMGB1 level was mitigated by iNOS deficiency (Fig 6).

**Fig 7:**
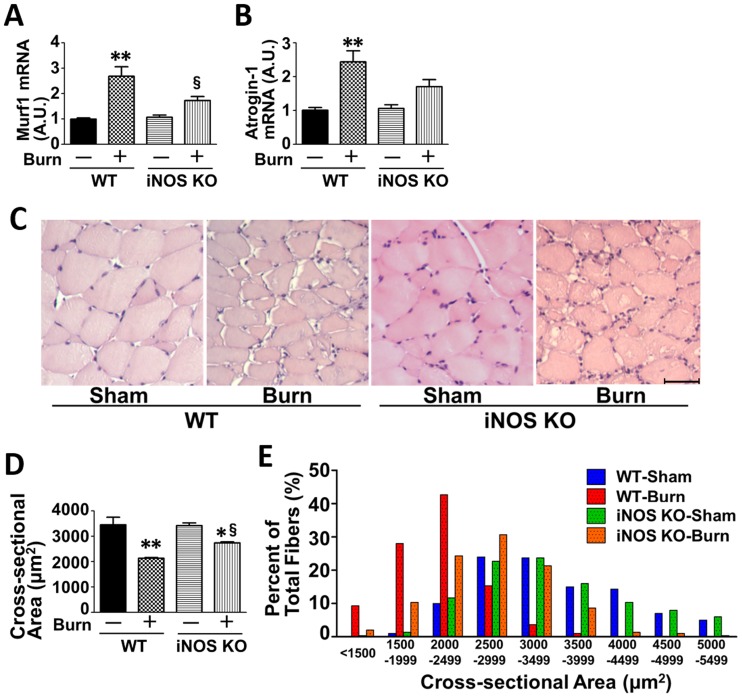
iNOS deficiency ameliorated increased expression of atrogenes and decreased cross-sectional area in skeletal muscle of burned mice. A, B, At 3 days after burn or sham-burn, mRNA levels of atrogenes, Murf1 and atrogin-1, were significantly increased by burn injury in wild-type (WT) mice. Burn-induced increase in mRNA levels of these genes was attenuated in iNOS knockout (iNOS KO) mice. n = 3 mice per group for Sham; n = 5 mice per group for Burn. C-E, Muscle fiber cross-sectional area was evaluated at 7 days after burn or sham-burn. Muscle fiber cross-sectional area was significantly decreased by burn injury. iNOS deficiency partially prevented burn-induced decrease in muscle fiber cross-sectional area. Scale bar: 50 μm. n = 3 mice per group. *P<0.05, **P<0.01 vs. WT-Sham and iNOS KO-Sham, §P<0.05 vs. WT-Burn.

To study the role of iNOS in burn-induced muscle wasting, we evaluated mRNA levels of muscle-specific ubiquitin ligases, Murf1 and atrogin-1, which are key enzymes for protein breakdown and muscle wasting. In wild-type mice, burn injury significantly increased mRNA expression of Murf1 and atrogin-1 at 3 days post-burn compared with sham-burn, both of which are downstream target genes of NF-κB [17, 18]. In contrast, burn failed to significantly increase Murf1 and atrogin-1 mRNA levels in iNOS knockout mice (Fig 7A and 7B). Consistently, burn decreased muscle fiber cross-sectional area in wild-type mice at 7 days post-burn compared with sham-burn. iNOS deficiency ameliorated burn-induced decrease in muscle fiber cross-sectional area. In sham-burned mice, iNOS deficiency did not affect none of these parameters (Fig 7C–7E).

## Discussion

**Fig 8:**
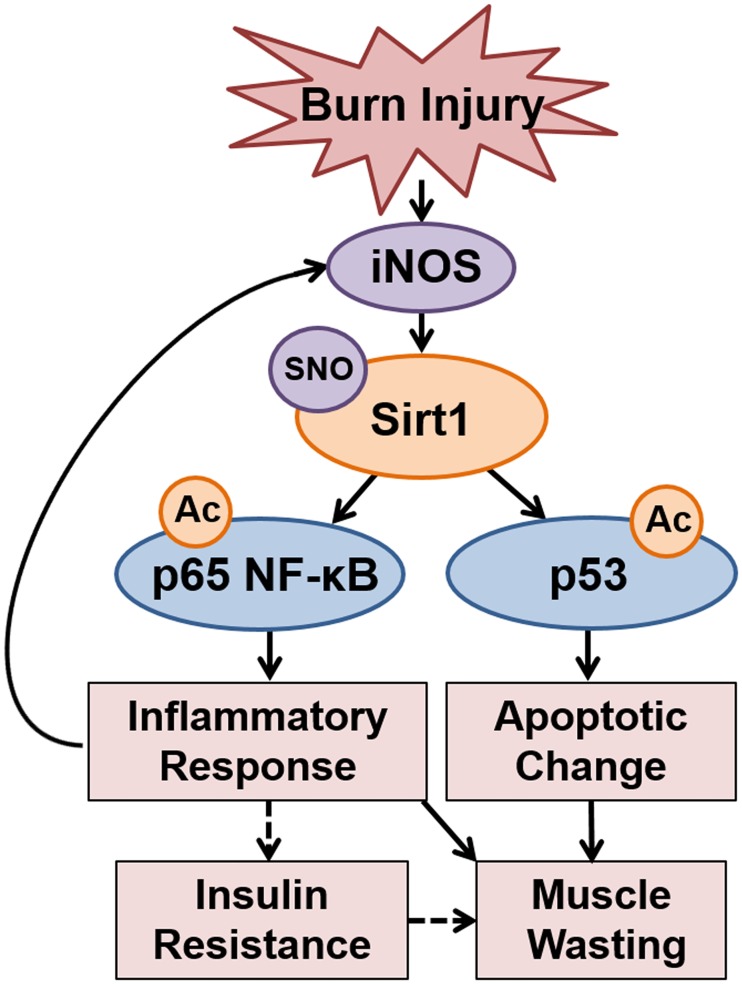
iNOS as a hub of burn-induced development of inflammatory response and apoptotic change. iNOS-dependent S-nitrosylation (SNO) of Sirt1 increases acetylation (Ac) and activation of p65 NF-κB and p53, which, in turn, induces and/or enhances to inflammatory response and apoptotic change in skeletal muscle after burn injury. iNOS functions as a nodal point of the development of inflammatory spinal comprised of iNOS → Sirt1 S-nitrosylation → acetylation (activation) of p65 NF-κB → iNOS, which, in turn, supposedly contributes to burn-induced insulin resistance and muscle wasting.

Here, we show that burn injury induced acetylation and activation of p65 NF-κB and p53 at 3 days post-burn, along with iNOS induction and S-nitrosylation of Sirt1 in mouse skeletal muscle. iNOS deficiency prevented these alterations in burned mice. Our data indicate that iNOS induction is required for Sirt1 S-nitrosylation and acetylation of p65 NF-κB and p53 after burn injury. Moreover, the inhibition of acetylation and activation of p65 NF-κB and p53 by iNOS deficiency were associated with amelioration of burn-induced inflammatory response and apoptotic change as well as the prevention of insulin resistance [13] (Fig 8).

iNOS is a major target gene of NF-κB. Conversely, iNOS is capable of both enhancing and inhibiting NF-κB activity [16]. In most, but not all, rodent models of inflammatory human diseases, including ischemia-reperfusion injury, inhibition of iNOS by gene disruption or pharmacological inhibitors decreases NF-κB activity [40–43]. However, the underlying mechanisms by which iNOS increases NF-κB activity were not fully understood. We have shown that S-nitrosylation-mediated inactivation of Sirt1 plays a pivotal role in iNOS-induced enhancement of NF-κB activity in cultured cells by increasing acetylation of p65 NF-κB [16]. Our data suggest that iNOS-mediated Sirt1 S-nitrosylation and associated acetylation (activation) of p65 NF-κB may be an important component of a positive feedforward loop in which iNOS functions as a driver of the development of inflammatory response after burn injury. It is important to note that our data do not exclude the possibility that signaling molecules other than Sirt1 may be involved in iNOS-mediated enhancement of NF-κB acetylation after burn injury.

Unlike iNOS, neither nNOS nor eNOS expression was increased after burn injury in wild-type and iNOS-deficient mice. These data indicate that iNOS, but not nNOS or eNOS, plays a major role in Sirt1 S-nitrosylation after burn injury.

iNOS deficiency almost completely prevented burn-induced acetylation of p65 NF-κB and p53 (Fig 2). In line with this, the induction of proinflammatory and proapoptotic genes was significantly attenuated by iNOS deficiency (Figs 4 and 5). The inhibition of the expression of these genes by iNOS deficiency, however, seems relatively modest compared with the almost complete inhibition of acetylation of p65 NF-κB and p53. These results suggest that over and above the iNOS→acetylation of p65 NF-κB and p53 pathway, some other alternate signaling pathways may be also involved in the proinflammatory and proapoptotic gene expression after burn, particularly in the absence of iNOS. Unlike acetylation, iNOS deficiency did not alter burn-induced phosphorylation of p65 NF-κB and p53, which increases the transcriptional activation of p65 NF-κB and p53. One can reasonably speculate that the relatively modest inhibition of the downstream gene expression of p65 NF-κB and p53 by iNOS deficiency may be at least in part attributable to the lack of the effects of iNOS deficiency on phosphorylation of p65 NF-κB and p53. It should be noted, however, that iNOS deficiency exerted significant protective effects on burn-induced apoptotic change and muscle wasting, as well as insulin resistance as previously described [13]. Together, our data indicate that the iNOS→acetylation of p65 NF-κB and p53 pathway has a biological relevance as a driver of the development of inflammation and apoptotic change in a mouse model of burn injury.

Our previous study has shown that iNOS is expressed in skeletal muscle cells after burn in mice [13]. It is reasonable to speculate that iNOS expressed in skeletal muscle cells contributes to the burn-induced S-nitrosylation of Sirt1 and subsequent acetylation of p65 NF-κB and p53. However, our data cannot exclude the possibility that iNOS expressed in infiltrated immune cells (e.g., macrophages) may also play a role in the Sirt1 S-nitrosylation and p65 NF-κB and p53 acetylation in mouse skeletal muscle.

Regarding the downstream genes of NF-κB, including TNF-α, IL-1β and iNOS, our findings are consistent with previous studies that burn injury increases these pro-inflammatory genes in skeletal muscle [13, 25, 44–47]. Previous studies have shown that TNF-α and IL-1β proteins are expressed in human and rodent skeletal muscle cells both under normal and disease conditions [48–51] and that iNOS protein is induced in mouse skeletal muscle after LPS injection [52, 53] and burn injury [13]. Moreover, it has been proposed that locally produced and secreted TNF-α by skeletal muscle cells, rather than circulating TNF-α, plays an important role in obesity-induced insulin resistance in skeletal muscle [54]. These previous studies support the biological relevance of pro-inflammatory gene expression in skeletal muscle cells.

Although skeletal muscle cells make up most of the cellular mass within skeletal muscle tissue, whole skeletal muscle homogenates, which were used for the evaluation of mRNA expression levels, represent a mixture of different cell types, including immune cells (e.g., macrophages, neutrophils) as well as skeletal muscle cells. Therefore, our data do not exclude the possibility that infiltrated immune cells (e.g., macrophages) may contribute to the increased expression of pro-inflammatory genes, such as TNF-α, IL-1β and iNOS. It is important to note that NO produced by iNOS in macrophages may be capable of inducing nitrosative stress in adjacent cells, which is exemplified by adipose tissue inflammation by macrophage iNOS in obesity-induced diabetes [55].

Muscle wasting is a clinically important complication of critical illness, including burn injury. Muscle wasting in the critical ill interferes with recovery, and prolongs mechanical ventilator-dependence, hospital stay and rehabilitation [56]. Increased protein breakdown, which exceeds protein synthesis, is a major contributor to critical illness-associated muscle wasting. Inflammatory response and insulin resistance play important roles in muscle wasting by increasing the expression of Murf1 and atrogin-1 [8, 9, 17, 18]. Our previous studies have shown that iNOS deficiency inhibits stress (e.g., burn injury)- and obesity-induced insulin resistance in skeletal muscle [12, 13]. In this study, iNOS deficiency mitigated increased expression of Murf1 and atrogin-1 and decreased muscle fiber size in burned mice. Moreover, apoptotic changes contribute to muscle atrophy [6, 7, 16]. Together, it is conceivable that inflammatory response, insulin resistance, and apoptotic change, all of which are aggravated by iNOS, may contribute in concert to burn-induced muscle wasting.

NF-κB can promote both cell survival and cell death [57], presumably dependent on degrees of NF-κB activation, patterns of target gene expression, and as yet identified cellular context. NF-κB activation after burn injury was associated with apoptotic change in mouse muscle, consistent with previous studies [24, 34]. Little is known about the underlying molecular mechanisms that dictate the impact of NF-κB activation on cell fate toward opposite directions, namely, cell survival and apoptosis. Moreover, the relationship between NF-κB and p53 is not well understood. Together with our previous studies [16], our findings raise the possibility that when concerted activation of p65 NF-κB and p53 is induced by iNOS-mediated S-nitrosylation and inactivation of Sirt1, it may tend to promote apoptotic change rather than cell survival. Further studies are required to clarify this possibility.

iNOS plays an important role in the development of obesity- and stress-induced insulin resistance in skeletal muscle [12–14]. However, it remains to be determined how iNOS induces and/or exacerbates insulin resistance in skeletal muscle. It is important to note that inflammatory response plays a critical role in the pathogenesis of insulin resistance [3–5]. Based on previous studies and our data, one can speculate that iNOS may contribute to the development of burn-induced insulin resistance, at least in part, by enhancing inflammatory response. Moreover, previous studies have shown that insulin resistance is capable of inducing muscle atrophy [9, 58, 59]. Together, it is possible that iNOS-induced enhanced inflammation may contribute to insulin resistance, thereby leading to exacerbation of burn-induced muscle wasting.

In summary, our data indicate that iNOS plays a critical role in burn-induced Sirt1 S-nitrosylation and acetylation and activation of p65 NF-κB and p53 in mouse skeletal muscle. These findings identify iNOS as a potential hub of the signaling network that orchestrates the development of inflammatory response and apoptotic change after burn injury, which, in turn, supposedly contribute to insulin resistance and muscle wasting (Fig 8).

## Supporting Information

S1 Fig Protein expression of p65 NF-κB, p53, GAPDH and histone H3 was not altered by burn injury. (TIF)

S2 Fig Protein Expression of p65 NF-κB, p53, Sirt1, GAPDH and histone H3 was not altered by burn injury or iNOS deficiency in mouse skeletal muscle at 3 days after burn or sham-burn. (TIF)
