# Supplementary figures and images for: iNOS as a Driver of Inflammation and Apoptosis in Mouse Skeletal Muscle after Burn Injury: Possible Involvement of Sirt1 S-Nitrosylation-Mediated Acetylation of p65 NF-κB and p53
Source: PLoS One. 2017 Jan 18;12(1):e0170391. doi: 10.1371/journal.pone.0170391 (PMC5242494; doi:10.1371/journal.pone.0170391)

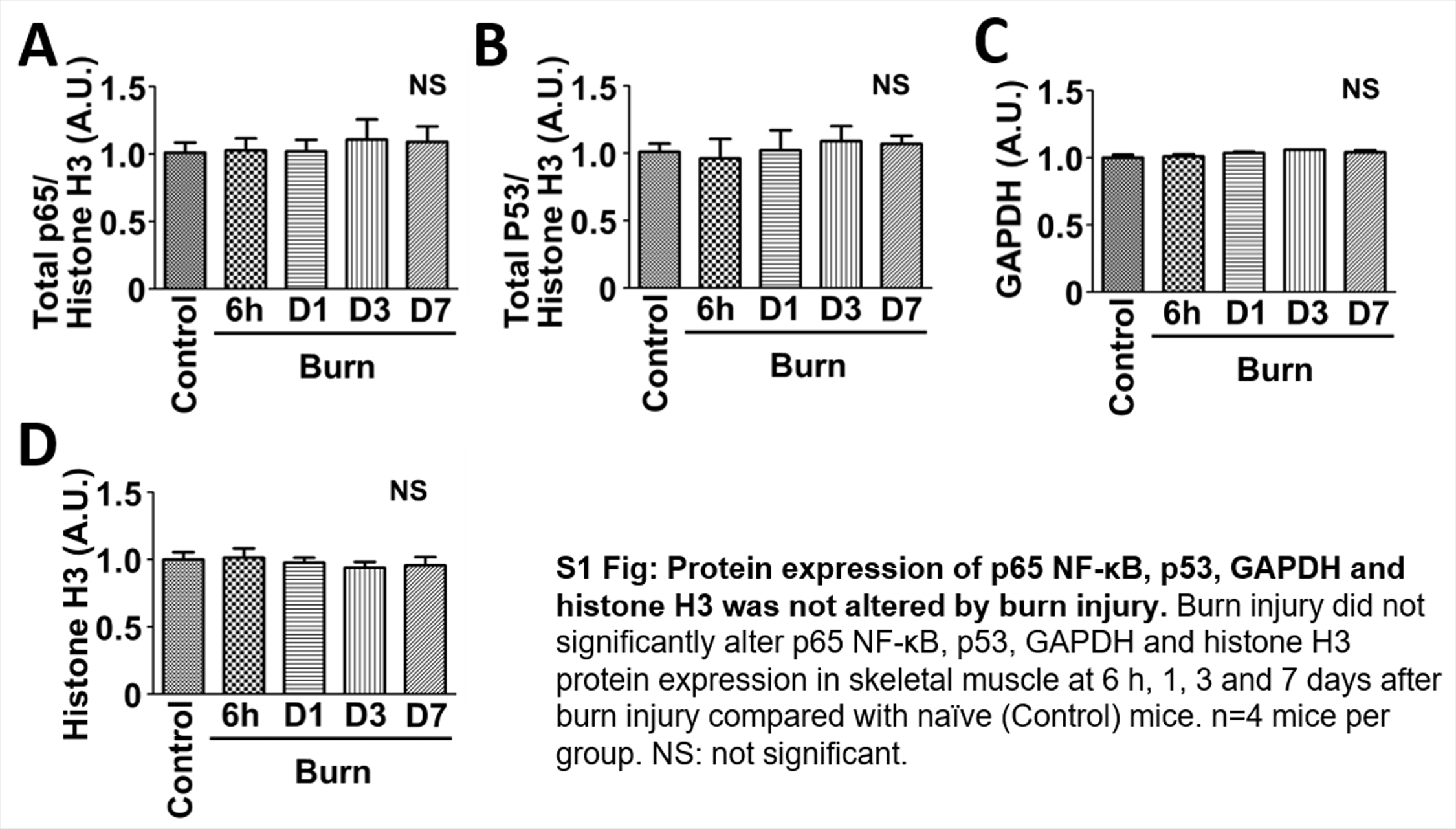

Supplement: S1 Fig — (TIF) [file pone.0170391.s001.tif]

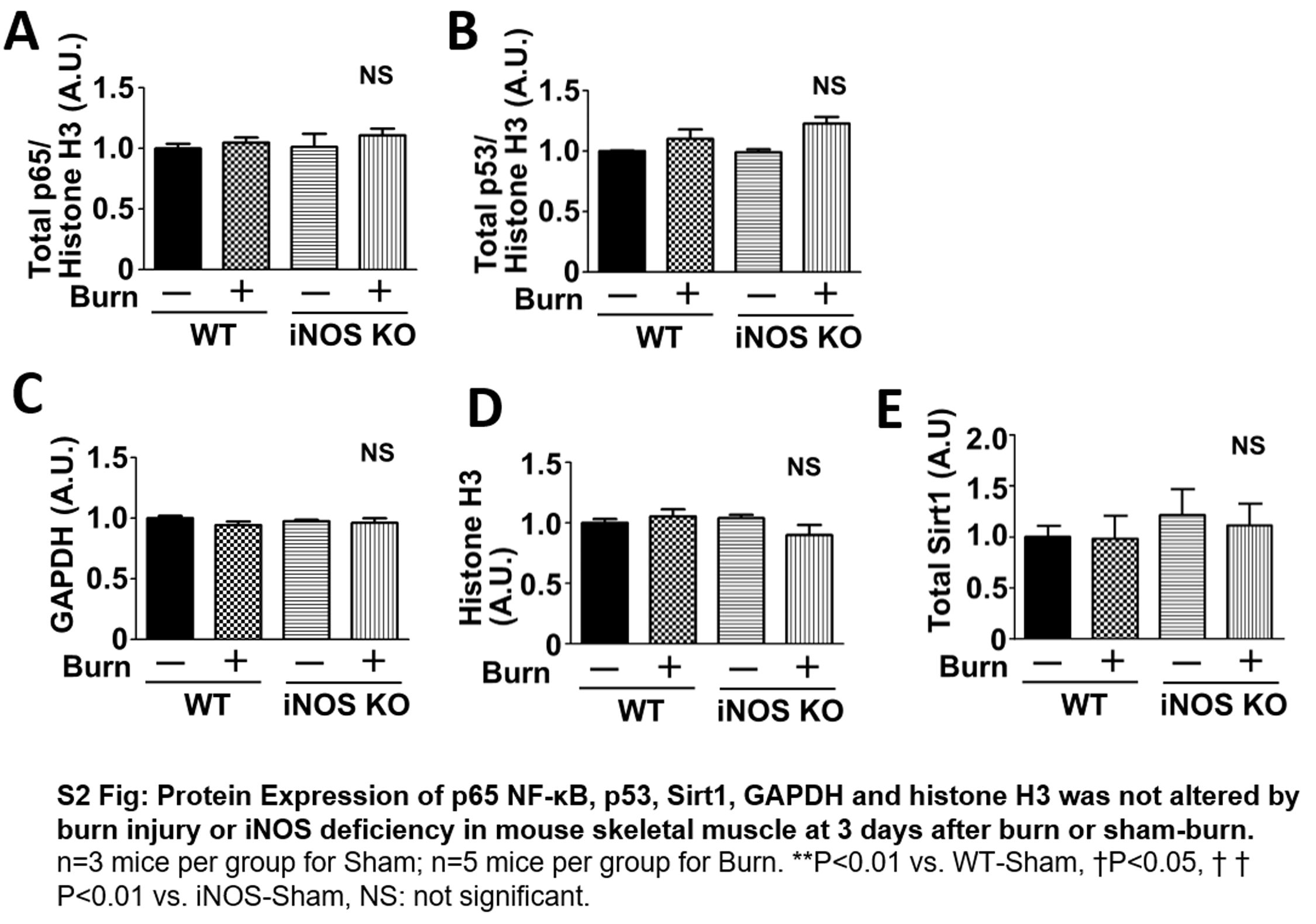

Supplement: S2 Fig — (TIF) [file pone.0170391.s002.tif]
